# Was It Glanders?

**Published:** 1884-04

**Authors:** R. Lowell

**Affiliations:** Potsdam, N. Y.


II.—WAS IT GLANDERS?

Some time in September, 1883, a bay mare was brought to my office affected (so owner said, he had only purchased her a few days before) with a bad cold. She presented a loathsome appearance, breathing very labored; a copious discharge of thick fetid pus from both nostrils, coat staring, but otherwise in fair condition, membrane of nostrils of a deep leaden hue, presenting a mouse eaten appearance. Inoculated her on neck and arm with pus from the nostril, making the smallest incision possible; sent her home saying I would call in four or five days. On calling, found in both places where inoculated a glary discharge of pus with considerable tumefaction; lymphatic glands closely adherent to jaws; stated my suspicion of her being glandered and strongly advised having her destroyed. Called about a week afterward and found the owner had disposed of her to a jockey who took her some twenty miles away and changed her off. Am watching for further developments and will communicate to the "Journal" any thing of interest arising therefrom.

POTSDAM, N. Y. R. LOWELL, V. S.

